# The role of lipid metabolism in osteoporosis: Clinical implication and cellular mechanism

**DOI:** 10.1016/j.gendis.2023.101122

**Published:** 2023-09-20

**Authors:** Jing Zhang, Wenhui Hu, Zhi Zou, Yuheng Li, Fei Kang, Jianmei Li, Shiwu Dong

**Affiliations:** a College of Bioengineering, Chongqing University, Chongqing 400044, China; b Department of Biomedical Materials Science, College of Biomedical Engineering, Army Medical University (Third Military Medical University), Chongqing 400038, China; c State Key Laboratory of Trauma and Chemical Poisoning, Army Medical University (Third Military Medical University), Chongqing 400038, China

**Keywords:** Bone homeostasis, Cross-organ communication, Lipid metabolism, Osteoclast, Osteoporosis

## Abstract

In recent years, researchers have become focused on the relationship between lipids and bone metabolism balance. Moreover, many diseases related to lipid metabolism disorders, such as nonalcoholic fatty liver disease, atherosclerosis, obesity, and menopause, are associated with osteoporotic phenotypes. It has been clinically observed in humans that these lipid metabolism disorders promote changes in osteoporosis-related indicators bone mineral density and bone mass. Furthermore, similar osteoporotic phenotype changes were observed in high-fat and high-cholesterol-induced animal models. Abnormal lipid metabolism (such as increased oxidized lipids and elevated plasma cholesterol) affects bone microenvironment homeostasis via cross-organ communication, promoting differentiation of mesenchymal stem cells to adipocytes, and inhibiting commitment towards osteoblasts. Moreover, disturbances in lipid metabolism affect the bone metabolism balance by promoting the secretion of cytokines such as receptor activator of nuclear factor-kappa B ligand by osteoblasts and stimulating the differentiation of osteoclasts. Conclusively, this review addresses the possible link between lipid metabolism disorders and osteoporosis and elucidates the potential modulatory mechanisms and signaling pathways by which lipid metabolism affects bone metabolism balance. We also summarize the possible approaches and prospects of intervening lipid metabolism for osteoporosis treatment.

## Introduction

Disorders of lipid metabolism are qualitative and quantitative changes in lipids and their metabolites in blood or other tissues, including total cholesterol, triglycerides, low-density lipoprotein (LDL) and high-density lipoprotein, phospholipids, fatty acids, and lipid oxides.^1^ Metabolic disorders, obesity, and insulin resistance are the risk factors for dyslipidemia and lipid metabolism disorders,^2^ producing many diseases associated with abnormal lipid metabolism, such as nonalcoholic fatty liver disease (NAFLD), atherosclerosis, and cardiovascular diseases. NAFLD and atherosclerosis frequently occur together with osteoporosis. It has been demonstrated clinically in humans that abnormalities of lipid metabolism cause changes in osteoporosis-related indicators like bone mineral density (BMD) and bone mass in diseases such as NAFLD and atherosclerosis. Consequently, it is essential to comprehend the role and mechanism of lipid metabolism in osteoporosis development.

Osteoporosis is a progressive bone disease characterized by bone mass loss and changes in the bone microstructure, affecting bone strength and increasing fracture risk.^3^ Osteoporosis is prevalent among the elderly and postmenopausal women.^4^ Risk factors for osteoporosis include aging, estrogen deficiency, lipid metabolism disorders, and obesity. The association between lipids and bone has received more attention in recent years. The disorder of lipid metabolism might be a major cause of osteoporosis, and in particular, many studies were conducted on oxidized lipids and plasma cholesterol. Oxidized lipids are modified lipids via oxidation and lipid metabolism disorders could lead to an increase in oxidized lipids.

Furthermore, oxidized lipids promote lipogenic differentiation of mesenchymal stem cells (MSCs) primarily by activating the peroxisome proliferator-activated receptor gamma (PPARγ) signaling pathway.^5^ Elevated plasma cholesterol levels down-regulate the Wnt signaling pathway, affecting MSC differentiation towards osteoblasts.^6^^,^^7^ Besides, oxidized lipids and cholesterol interfere with the balance of osteogenic and osteoclastic bone remodeling. Oxidized lipids stimulate osteoblasts to secrete receptor activator of nuclear factor-kappa B ligand (RANKL), causing excessive osteoclasts differentiation and bone resorption.^8^ Cholesterol binds with Smoothened protein and activates the Hedgehog signaling pathway, inhibiting osteoblast differentiation.^9^^,^^10^ Lipid metabolism disorders can affect bone microenvironment homeostasis, resulting in osteoporosis.

In this review, we discussed the potential link between lipid metabolism disorders and osteoporosis and elucidated the potential molecular mechanisms and signaling pathways by which lipid metabolism affects the occurrence and development of osteoporosis. This study may contribute to developing novel preventative medications, therapeutics, and predictive value for osteoporosis treatment. Finally, we summarized several strategies for preventing and treating osteoporosis, including diet and exercise, lipid interventions, and molecular targeted therapy. This review will add to our understanding of the function of lipid metabolism in osteoporosis and present innovative concepts for the therapeutic development of osteoporosis.

## Adverse effects of lipid metabolic diseases on bone

### Clinical findings

#### Osteoporosis phenotype in nonalcoholic fatty liver disease

NAFLD is a metabolic liver disease characterized histologically by the accumulation of triglycerides in hepatocytes, which can progress from mild hepatic steatosis to nonalcoholic steatohepatitis with liver inflammation and cirrhosis.^11^^,^^12^ Evidence suggests that free fatty acids and cholesterol are involved in key metabolic and inflammatory pathways in the pathogenesis of NAFLD; free fatty acids are a major source of triglycerides in the liver, and free cholesterol levels can lead to nonalcoholic steatohepatitis.^13^, ^14^, ^15^ Furthermore, triglycerides can be packaged into very-low-density lipoprotein in addition to accumulating in lipid droplets; increased very-low-density lipoprotein secretion due to high lipid content in the liver is a chief reason for dyslipidemia associated with NAFLD.^15^ In conclusion, hepatic steatosis, caused by lipid apoptosis, disrupts systemic metabolism and adversely affects various organs.^16^

Given the rising prevalence of NAFLD and osteoporosis and their propensity to occur in the elderly, osteoporosis is anticipated to be an important complication of NAFLD. Recent epidemiological studies confirmed that the incidence of osteoporosis is significantly higher in patients with liver disease than in those without liver disease.^17^ Li et al^18^ explored whether NAFLD was associated with osteoporotic fractures by selecting 7797 adults aged 40 or older in the Jiading district of Shanghai, China. They conducted a questionnaire to collect the fracture history of the NAFLD patients and reported that the prevalence of osteoporotic fractures was significantly higher in men with NAFLD than those without NAFLD, but no difference was found in women. In another study examining the correlation between liver fat content and serum alanine aminotransferase and BMD in an elderly population, researchers used dual-energy X-ray bone densitometry to measure lumbar spine, hip, and whole-body BMD in 1659 adults (755 men and 1028 postmenopausal women) in the Changfeng community of Shanghai. The results found that subjects with higher liver fat content had significantly lower BMD at all skeletal sites and that BMD at all bone sites was significantly and synergistically worse when NAFLD and alanine aminotransferase levels were elevated together.^19^ However, the above study had some limitations and might not apply to the entire population. Moreover, the main pathological and physiological factors of NAFLD impacting bone density and bone mass that led to osteoporosis include hormones, inflammatory factors, proteins, and lipids. Among them, lipids comprise vitamin D, triglyceride, cholesterol, *etc.*^4^ Overall, NAFLD may have a negative effect on bone mass and BMD, which in turn promotes osteoporosis.

#### Atherosclerosis and osteoporosis in humans

Atherosclerosis is a chronic inflammatory disease characterized by lipid accumulation in the artery wall, and the major hallmarks are lipid metabolism disorders and hypercholesterolemia.^20^ The main source of lipids accumulating in atherosclerotic plaques is circulating LDL.^21^ Furthermore, under oxidative stress, polyunsaturated fatty acid phospholipids and cholesterol esters contained in cell membranes and lipoproteins can form complex lipid oxidation products through free radical-induced lipid peroxidation, and these oxidized lipids can impair normal physiological functions and stimulate atherogenesis.^22^^,^^23^ Moreover, the evidence suggests osteoporosis and atherosclerosis have the same risk factors or pathophysiological processes, implying that the occurrence of atherosclerosis and osteoporosis may be related.

Possible common risk factors for atherosclerosis and osteoporosis include aging, estrogen deficiency, dyslipidemia, oxidized lipid products, diabetes, hypertension, and chronic inflammation.^24^^,^^25^ In a study cohort of Finnish young adults based on cardiovascular risk followed in 2007, investigators used liquid chromatography-tandem mass spectrometry to analyze the serum lipidome to identify tightly linked lipid species associated with subclinical markers of osteoporosis and atherosclerosis. The results showed that the lipid module was significantly associated with subclinical markers of osteoporosis and atherosclerosis, and among the 37 significantly associated lipids, the top three were triglycerides (18:0/18:0/18:1), triglycerides (18:0/18:1/18:1), and triglycerides (16:0/18:0/18:1).^26^ Besides, glycerophospholipids, sphingolipids, and cholesterol are also included.^26^ Furthermore, in a study examining the relationship between atherosclerosis and BMD in 332 men without a history of cardiovascular disease, researchers assessed atherosclerosis and BMD-related metrics; among the healthy middle-aged and older men without a history of cardiovascular disease, the positive association between atherosclerosis and BMD was explained primarily through body weight, of which body fat mass was a component, and that excess fat could promote osteoporosis.^27^ Research also demonstrates that among the prevalent risk factors for atherosclerosis and osteoporosis, lipid oxidation products such as minimal oxidized LDL promote arterial calcification and their accumulation in the subendothelial space of skeletal arteries inhibiting bone formation. To summarize, these results revealed that lipids play a crucial role in atherosclerosis and osteoporosis development.

#### Obesity-induced osteoporosis

Obesity is a worldwide epidemic. According to the World Health Organization, overweight and obesity are considered abnormal or excessive fat accumulation that can be detrimental to health. Obesity or excess weight increases the risk of coronary artery diseases, hypertension, type 2 diabetes (T2D), and osteoporosis.^28^, ^29^, ^30^ Furthermore, dyslipidemia due to obesity is characterized by high levels of triglyceride-rich lipoproteins and low levels of high-density lipoprotein cholesterol (HDL-C).^31^ In addition, a study assessing obesity and lipid levels in 10-year-old children over 27 years found that obese children had significantly higher total cholesterol, triglyceride, and non-high-density lipoprotein cholesterol levels and lower high-density lipoprotein levels than non-obese children.^32^ Most clinical studies have shown the association between obesity and osteoporosis risk.

A trial on the effects of obesity on bone metabolism, microarchitecture, and strength in 112 obese male patients with and without type 2 diabetes assessed bone turnover and biochemical markers and compared data between obese patients with and without type 2 diabetes. The results showed that obese men with type 2 diabetes had significantly lower osteocalcin and carboxy-terminal collagen cross-linking levels and poorer bone trabecular microarchitecture and strength; obesity and type 2 diabetes resulted in severe bone diseases.^33^ In addition, low muscle and high fat give rise to bone function defects and poorer bone health, increasing the risk of falls and fractures, and osteoporosis is prevalent in people with low muscle mass.^34^ Leslie et al^35^ studied bone health as a function of estimated total lean and fat mass in 40,050 women and 3600 men under 50 and measured femur and tibia bone density, strength index, cross-sectional area, and other metrics. According to the findings, the increased fat mass did not influence bone density but had a negative effect on femur strength, predicting a higher chance of fracture.

#### Osteoporosis phenotype in postmenopausal women

Female menopause is the natural cessation of menstruation due to loss of follicular activity, and surgical menopause is also accompanied by loss of estradiol.^36^ Thus, the distinguishing feature of a naturally or surgically menopausal woman is hormonal changes, such as a decrease in estrogen, a rise in follicle-stimulating hormone (FSH), and an increase in circulating androgens.^37^ It has been shown that the rise in FSH in menopausal women contributes to adiposity and dyslipidemia. Epidemiologic studies have shown that high FSH levels are positively correlated with total cholesterol and low-density lipoprotein cholesterol (LDL-C) in menopausal women, especially young menopausal women (588 subjects aged 53–73 years).^38^ As the prevalence of osteoporosis increases in postmenopausal women,^39^ it is therefore crucial to study the mechanisms of osteoporosis in menopausal women.

In a study evaluating the relationship of antioxidant enzymes and lipid peroxidation products to BMD and bone mass in 87 postmenopausal women (aged 40–65 years without a history of osteoporosis), the investigators concluded from measurements of BMD and related metrics that lipid peroxides may be a key indicator of bone loss in postmenopausal women.^40^ In addition, Yoshida et al^41^ explored the effects of ovariectomy on lipid and bone metabolism (62 women, divided into an ovary-preserved group and a bilateral oophorectomy group) and demonstrated that ovariectomized women had significantly higher LDL levels and significantly lower BMD levels. The lack of hormones in menopausal women causes an increase in bone marrow-derived adipocytes, resulting in fat redistribution, which leads to an increase in visceral fat in postmenopausal women, and lipolysis of visceral fat produces large amounts of free fatty acids.^37^ Mao et al^42^ showed that FSH binds to the follicle-stimulating hormone receptor, which activates the classical cyclic adenosine monophosphate (cAMP)/protein kinase A (PKA)/cAMP response element-binding protein pathway, and increased adipocytic PPARγ transcription promotes the production of lipids and a range of lipogenic factors. Therefore, since menopausal women are often accompanied by pathological manifestations such as dysregulation of lipid metabolism and obesity, the development of osteoporosis in menopausal women may perhaps be related not only to estrogen deficiency but also to disorders of lipid metabolism.

### Osteoporosis in animal models of a high-fat diet

The osteoporosis phenotype has been noted in several animal models under conditions of disturbed lipid metabolism, suggesting that disturbed lipid metabolism adversely affects the development and course of osteoporosis. Some investigators have studied the phenotype of osteoporosis in a number of high-fat-fed animal models. Investigators assessed the effect of micro-oxidized LDL on osteoblast differentiation and its ability to regulate lipogenesis in mouse bone marrow stromal cells (M2-10B4) after feeding a high-fat and atherogenic diet to C57BL/6 mice and showed that oxidized lipids such as minimal oxidized LDL inhibit the differentiation of MSCs to osteoblasts and promote lipogenic differentiation, thereby promoting osteoporotic bone loss.^43^ In another study, You et al^6^ explored the effect of a high-cholesterol diet on osteoporosis development in rats and its mechanism by establishing a rat model fed with a high-cholesterol diet. They measured BMD, osteocalcin, carboxy-terminal collagen cross-linking, and gene expression profiles and observed the changes in these indicators and differentiation of osteoblasts in rats. The study reported that femoral BMD and serum osteocalcin levels were significantly lower, and carboxy-terminal collagen cross-linking levels were significantly higher in rats fed a high-cholesterol diet. Likewise, the expression of genes related to bone formation, such as transforming growth factor β/bone morphogenetic protein and Wnt signaling pathway-related proteins, was reduced in rats fed a high-cholesterol diet; the expression of genes related to bone resorption was increased, such as interleukin-6 and Ager. Furthermore, in one study, researchers selected two strains of mice with different susceptibilities to atherosclerosis and examined the effects of an atherogenic high-fat diet and a control diet on the bones of the mice. After four and seven months of the diet, they measured the mineral content and density of the resected femur and lumbar spine and examined the expression of osteocalcin in the bone marrow of the mice after four months of feeding. The results showed that the high-fat diet reduced the mineral content of femurs in atherosusceptible mice by 43%, the mineral density was 15% lower than that of mice on a normal diet, and that osteocalcin expression was reduced in the bone marrow of atherosclerotic mice fed a high-fat diet. That is, an atherogenic high-fat diet can inhibit bone formation.^44^ In summary, hyperlipidemia or hypercholesterolemia is closely associated with osteoporosis, and a high-cholesterol diet increases the osteoporosis risk.

### Interorgan communication of bone

**Figure 1:**
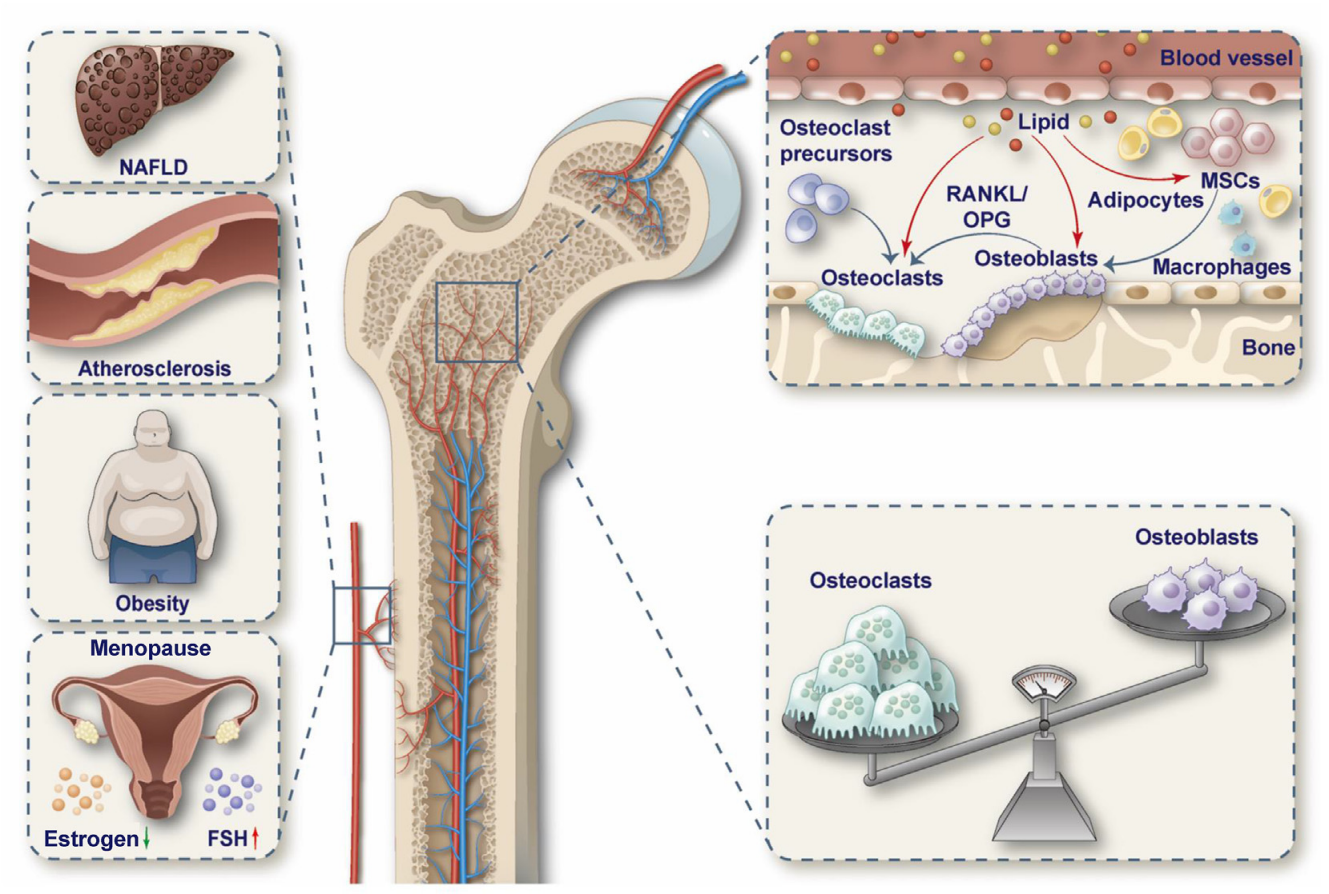
The effect of lipid metabolic diseases on osteoporosis. Many diseases, such as NAFLD, atherosclerosis, obesity, and postmenopause can cause lipid metabolism disorders in the body. Lipid metabolism will affect bone metabolism through blood circulation, mainly including lipid promotion of lipogenic differentiation of MSCs and inhibition of osteoblast differentiation. This makes bone resorption greater than bone formation, leading to osteoporosis. NAFLD, nonalcoholic fatty liver disease; MSCs, mesenchymal stem cells; FSH, follicle-stimulating hormone; RANKL, receptor activator of nuclear factor-kappa B ligand; OPG, osteoprotegerin.

Bone is a paracrine and an endocrine organ; bone-derived secretory factors regulate the normal physiological functions of other organs to ensure material balance and health in the body.^45^ Substances released by other vital metabolic organs, in turn, can influence bone formation and development. Thus, bone has a highly common cross-organ connection, and signaling axes exist between other organs and bone that govern their respective functions.^46^ The liver–bone axis and the bone–vascular axis are currently being researched in depth (Fig. 1).

#### Liver–bone crosstalk

The liver is a metabolic center that regulates the homeostasis in the body by interacting with multiple organs. Chronic liver disease patients frequently exhibit reduced BMD, defined as hepatic osteodystrophy,^47^ which is a classic example of the liver as a metabolic organ affecting bone metabolism. Nussler et al^47^ treated C57BL/6 mice with CCL4 for six weeks to establish a liver injury model. The μCT analysis showed a significant decrease in BMD, bone volume, and trabecular number and thickness in the CCL4-treated mice. Serum analysis disclosed decreased 25-OH vitamin D levels and excessive active transforming growth factor β, inhibiting osteoblasts function *in vitro*. It has been demonstrated that the expression of the intrahepatic phosphatase PP2Aca is up-regulated during hepatic osteodystrophy, leading to the down-regulation of the expression of the hepatic factor lecithin cholesterol acyltransferase; the loss of lecithin cholesterol acyltransferase's function significantly exacerbates the bone loss phenotype in mice with hepatic osteodystrophy.^46^ In addition, hepatocyte-derived insulin-like growth factor binding protein 1 enhances the transcriptional activity of nuclear factor-activated T cell 1 in osteoblasts by binding to the receptor integrin β1 on osteoblast precursors, promoting osteoblast differentiation and bone resorption.^48^

#### Bone–vascular crosstalk

The vasculature is a multifunctional transport network that plays a key role in tissue oxygenation, substance metabolism, and immune detection. In the skeletal system, the local vascular system has an active role in bone formation and resorption, and thus the connection between cardiovascular disease and bone is particularly strong.^49^ Bone is a mineral reservoir required for vascular integrity. Furthermore, platelet-derived growth factor-BB secreted by the skeleton is a major source of its excess in the circulation during aging and metabolic stress; skeletal-derived platelet-derived growth factor-BB is an important mediator of vascular sclerosis.^50^^,^^51^ The liver and bone or the blood vessels and bone can influence their respective metabolism through substance exchange. Therefore, lipid metabolism abnormalities caused by NAFLD and atherosclerosis may influence bone metabolism via the liver–bone or the vascular–bone axis. Recognition of these tissue axes will broaden our understanding of the pathogenesis of skeletal-related metabolic disorders and degenerative diseases.

## Mechanism of oxidized lipids and cholesterol affecting bone metabolism

### Oxidized lipids and cholesterol affect MSC differentiation

**Figure 2:**
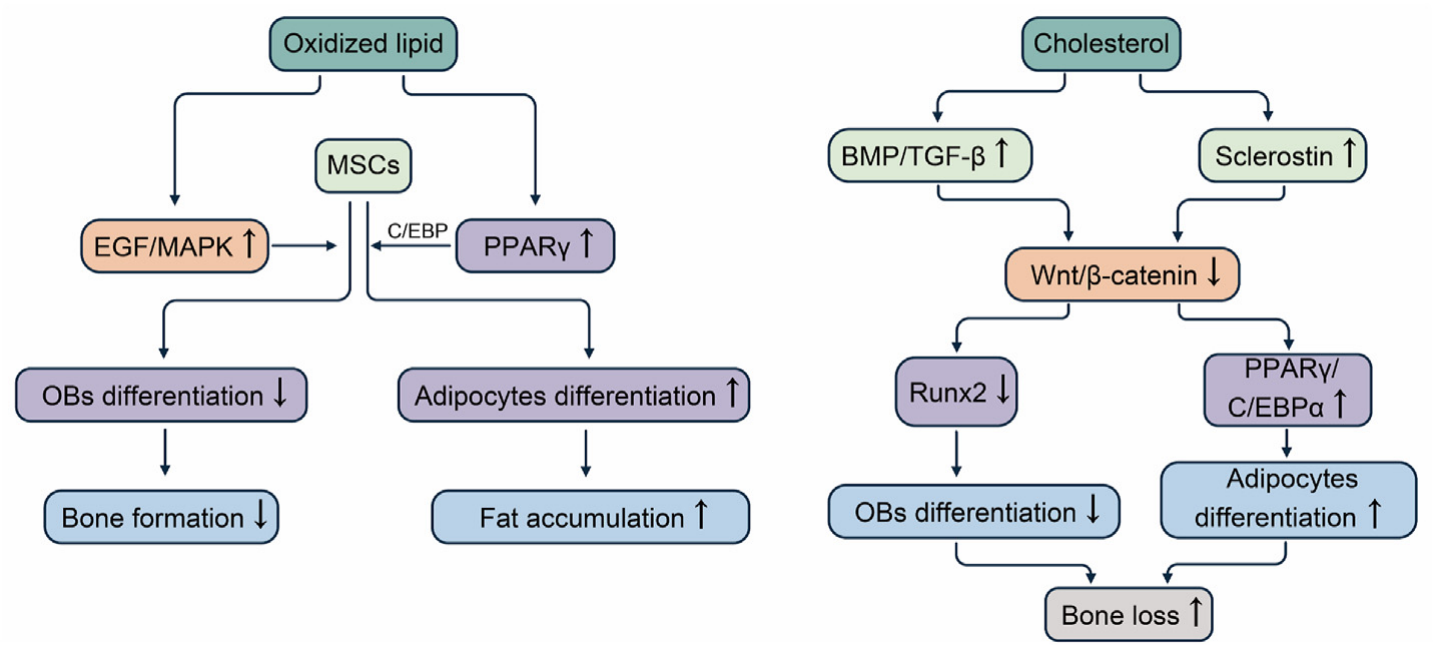
Oxidized lipids and cholesterol affect MSC differentiation. Oxidized lipids bind to and activate the PPARγ/C/EBP signaling pathway, promoting adipocyte differentiation, leading to fat accumulation. It also activates the EGF/MAPK pathway in MSCs and inhibits osteoblast differentiation. Cholesterol activates the BMP/TGF-β pathway and increases serum sclerostin levels, which leads to down-regulation of the Wnt/β-catenin pathway. Low activity of the Wnt pathway down-regulates Runx2 expression, thereby inhibiting osteogenic differentiation. The low-activity Wnt pathway also up-regulates PPARγ and C/EBPα to enhance adipocyte differentiation. MSCs, mesenchymal stem cells; PPARγ, peroxisome proliferator-activated receptor gamma; C/EBP, CCAAT/enhancer binding protein; EGF, epidermal-like growth factors; MAPK, mitogen-activated protein kinase; BMP, bone morphogenetic protein; TGF-β, transforming growth factor β; Runx2, runt-related transcription factor 2.

Bone marrow-derived MSCs are a multispectral cell lineage that can self-renew and differentiate into multiple cell types to maintain skeletal homeostasis.^52^ Adipogenesis and osteogenesis are the two opposite directions of MSC differentiation, and biological, physical, and chemical factors regulate the specific direction of differentiation; the balance of MSC differentiation is affected in different bone marrow microenvironments where lipogenic-inducing factors inhibiting osteoblasts formation and osteogenic-inducing factors blocking adipogenesis.^3^ The development of age-related osteoporosis is accompanied by decreased bone formation and increased bone marrow fat accumulation. In osteoporosis, MSCs have a decreased ability to differentiate into osteoblasts and an increased capacity to differentiate into adipocytes, leading to decreased bone formation.^53^ There is evidence that lipids influence MSC differentiation via numerous signaling pathways; thus, lipid metabolism may have a role in the development and progression of osteoporosis (Table 1 and Fig. 2).

**Table 1:** Oxidized lipids and cholesterol influence osteoporosis in the cellular and molecular mechanisms.

Lipid	Lipid's function	Mechanism	References
Oxidized lipid	Promote the differentiation of mesenchymal stem cells into lipoblasts and inhibit osteogenic differentiation	Activate the PPARγ signaling and up-regulate C/EBP expression; activate the EGF/MAPK pathway	5,43
	Break the homeostasis between osteoblasts and osteoclasts	Act on the receptor EP1/TP on osteoblasts, activate the cAMP–PKA pathway, and promote the release of IL6/RANKL from osteoblasts; act on the receptor EP2/DP on osteoclasts and promote osteoclast differentiation through the cAMP–PKA pathway	8,64
	Induce bone marrow adipocyte senescence	Activate the PPARγ signaling and induce the SASP in bone marrow adipocytes	71
	Activate endothelial progenitor cells' autophagic flux and inhibit their proliferation	Increase the expression of STIM1 and intracellular Ca^2+^ concentration	81
Cholesterol	Promote the differentiation of mesenchymal stem cells into lipoblasts and inhibit osteogenic differentiation	Down-regulate the bone morphogenetic protein/TGF-β pathway and up-regulate plasma sclerostin level and thus down-regulate the Wnt/β-catenin pathway; inhibit Runx2 expression and promote PPARγ/C/EBPα expression due to low-activity Wnt pathway	5, 6, 7,57,58
	Break the homeostasis between osteoblasts and osteoclasts	Act on Smoothened protein and activate Hedgehog signaling pathway; act on liver X receptors and up-regulate RANKL/OPG level	9,10,68,69
	Impair autophagy and polarize towards M1 macrophages	Inhibit the autophagy-related 5 expression	76

Notes: PPARγ, peroxisome proliferator-activated receptor gamma; C/EBP, CCAAT/enhancer binding protein; RANKL, receptor activator of nuclear factor-kappa B ligand; PKA, cAMP-dependent protein kinase; TGF-β, transforming growth factor β; Runx2, runt-related transcription factor 2; OPG, osteoprotegerin; SASP, senescence-associated secretory phenotype; STIM1, stromal interaction molecule 1; EGF, epidermal-like growth factors.

#### Oxidative lipids activate the PPARγ signaling

PPARγ is a nuclear receptor and a master regulator of adipogenesis that regulates the transcription of adipocyte-specific genes through ligand binding activation.^54^ Oxidized lipids bind to and activate the PPARγ signaling pathway, and PPARγ activation acts synergistically with CCAAT/enhancer binding protein to differentiate MSCs along the lipogenic pathway, while inhibiting osteoblast differentiation, leading to fat accumulation and inhibiting bone formation.^5^ In addition, some oxidized lipids induce the expression of epidermal-like growth factors by acting on their receptors in bone marrow stromal cells, thereby activating the mitogen-activated protein kinase pathway and inhibiting alkaline phosphatase activity and osteoblast differentiation in bone marrow stromal cells.^43^

#### Cholesterol abnormalities down-regulate the Wnt pathway

The Wnt signaling pathway is vital for regulating bone homeostasis, and the classical Wnt-β-catenin signaling pathway is triggered by the binding of Wnt ligands to membrane receptors, including LDL receptor-related protein 5/6 and coiled-coil proteins, which in turn trigger intracellular cascade responses that regulate the differentiation of MSCs to osteoblasts and adipocytes.^55^^,^^56^ Low activity of the Wnt-β-catenin signaling pathway in MSCs enhanced adipocyte differentiation by up-regulating CCAAT/enhancer binding protein α, and PPARγ leads to fat accumulation while inhibiting osteoblast differentiation by down-regulating runt-related transcription factor 2 (Runx2) expression, thereby suppressing bone formation.^5^ Runx2 is a key transcription factor involved in osteoblast differentiation. Cholesterol can alter Runx2 levels by altering the bone morphogenetic protein/transforming growth factor β/Wnt signaling pathway involved in bone formation, impacting the MSCs' differentiation to osteoblasts and proliferation and maturation of osteoblasts.^6^^,^^7^ In addition, the investigators found that serum sclerostin levels were positively correlated with LDL cholesterol levels and that high blood cholesterol levels elevate circulating serum sclerostin levels, which act as antagonists of Wnt ligands and down-regulate the Wnt-β-catenin signaling pathway.^57^^,^^58^ Therefore, cholesterol could stimulate lipogenic differentiation of MSCs while inhibiting osteoblast differentiation, leading to increased bone fat deposition, decreased bone mass, and the development of osteoporosis.

### Effects of oxidized lipids and cholesterol on osteoblasts and osteoclasts

**Figure 3:**
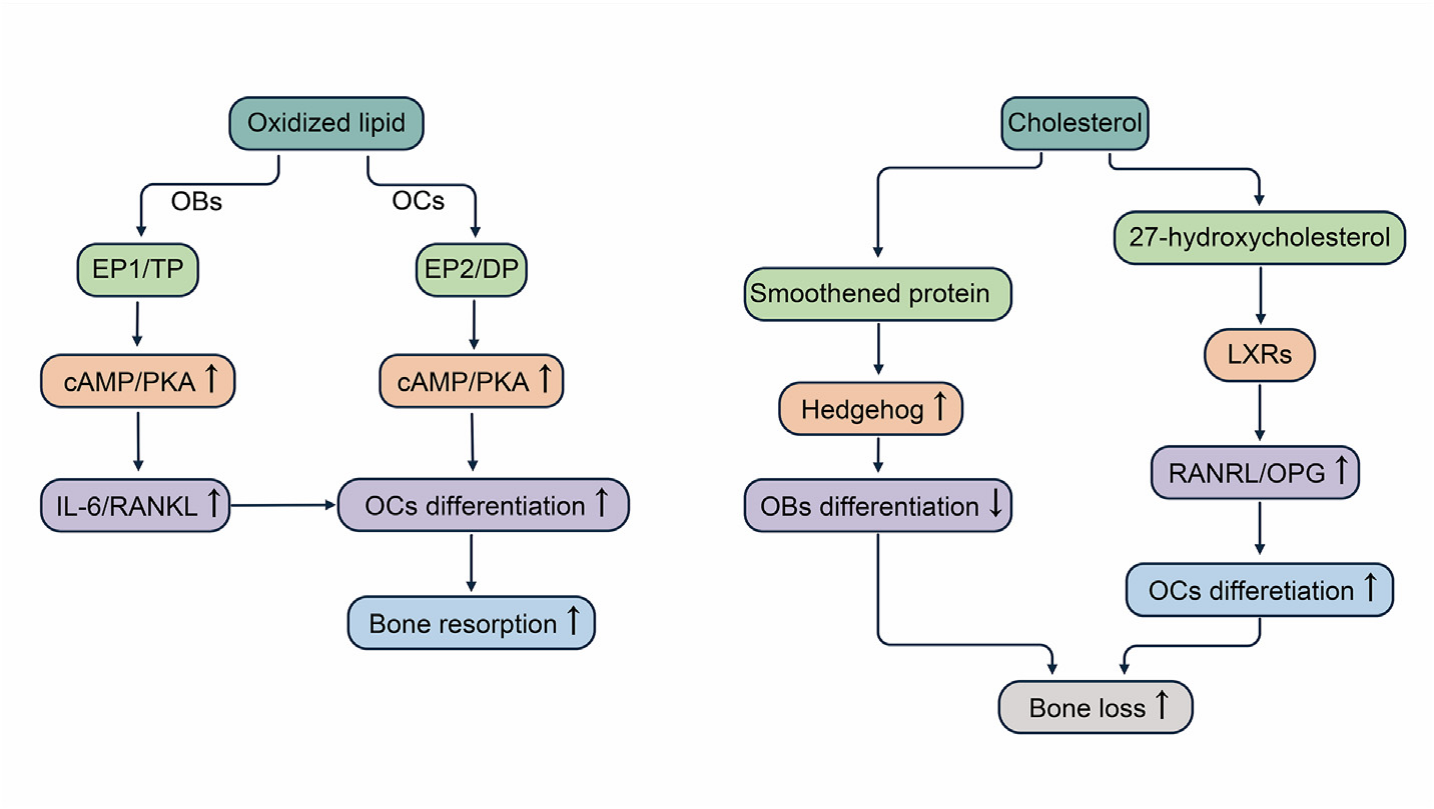
Oxidized lipids and cholesterol break the bone homeostasis between osteogenesis and osteolysis. Oxidized lipids act on the receptor EP1/TP on osteoblasts to induce the cAMP/PKA pathway in osteoblasts, leading to the release of cytokines. Oxidized lipids also act directly on EP2/DP receptors on osteoclasts to promote osteoclast differentiation via the cAMP/PKA pathway. Cholesterol binds to Smoothened protein and activates the Hedgehog signaling pathway, thereby inhibiting osteoblast differentiation. The intermediate metabolite of cholesterol, 27-hydroxycholesterol, acts on liver X receptors (LXRs) and increases the RANKL/OPG ratio, leading to the inhibition of osteoclast differentiation. PKA, cAMP-dependent protein kinase; RANKL, receptor activator of nuclear factor-kappa B ligand; OPG, osteoprotegerin.

Osteoblasts are mainly derived from MSCs inside and outside the periosteum and bone marrow stroma, and multinucleated osteoclasts differentiate from myeloid cells at different stages of maturation.^3^ The homeostasis of bone mass within healthy adults requires strict control of bone resorption by osteoclasts and bone formation by osteoblasts, and imbalance of bone formation and resorption is the underlying cause of various skeletal diseases including osteoporosis.^59^^,^^60^ Osteoblasts and osteoclasts communicate with each other mainly through direct cell-to-cell contact or secretion of cytokines to regulate cell growth and differentiation; for example, osteoblasts secrete macrophage colony-stimulating factor and RANKL/osteoprotegerin (OPG) to promote or inhibit the differentiation of osteoclasts.^61^ Osteoporosis results from an imbalance in the bone remodeling process through increased osteoclast activity and decreased osteoblast activity.^62^ Several studies have found that lipids can influence the differentiation and maturation of osteoclasts and osteoblasts, disrupting bone homeostasis. Understanding the mechanisms of lipid metabolism of osteoclasts and osteoblasts is thus critical for osteoporosis prevention (Fig. 3).

#### Oxidized lipids stimulate the cAMP–PKA cascade

RANKL plays a critical role in osteoclast proliferation and differentiation by binding to RANK receptors located on osteoclast precursor cells; it activates the mitogen-activated protein kinase signaling pathway, triggering various transcription factors and osteoclast differentiation markers such as nuclear factor kappa B, activator protein-1, and nuclear factor-activated T cell 1 downstream, thereby promoting osteoclast differentiation and bone resorption function.^63^ It has been shown that oxidatively modified bioactive lipids stimulate the production of interleukin-6 and RANKL by osteoblasts and T lymphocytes, inducing the cAMP–PKA pathway in osteoblasts by acting on their receptor EP1/TP, releasing these cytokines. Subsequently, overexpression of interleukin-6 and RANKL promotes osteoclasts over differentiation, encouraging bone resorption.^8^ On the other hand, oxidized lipids directly act on the prostaglandin receptor subtype EP2/DP on osteoclasts, increasing the level of cAMP in osteoclasts and ultimately promoting osteoclast differentiation via the PKA pathway.^64^

#### Cholesterol activates Hedgehog and LXR signaling

The Hedgehog signaling pathway is essential for osteoblast differentiation and plays an important role in bone homeostasis, and reducing Hedgehog signaling prevents age-related bone loss.^65^, ^66^, ^67^ Cholesterol binds to Smoothened proteins and activates the Hedgehog signaling pathway.^9^ Li et al^10^ investigated the role of exogenous and endogenous cholesterol in osteogenesis and Hedgehog pathway activation using a bone marrow stromal cell line (ST2 cells). The results found that exogenous cholesterol significantly inhibited alkaline phosphatase activity and osteoblast marker gene expression and moderately activated Hedgehog signaling pathway activity under basal osteogenic culture conditions, inhibiting the osteogenic differentiation of ST2 cells. In addition, using molecular and pharmacological approaches, Nelson et al^68^ demonstrated that the major metabolite of cholesterol, 27-hydroxycholesterol, may act on liver X receptors (LXRs). LXRs are a regulator of RANKL expression and RANKL/OPG ratio in osteoblasts. They can reduce osteoblast differentiation and activity, increase osteoclastic factors (tumor necrosis factor and RANKL), enhance osteoclast differentiation, and thus increase bone resorption in mice.^69^

### Effects of oxidized lipids and cholesterol on other cells in the bone microenvironment

Bone marrow adipocytes are an important class of cells in the bone marrow microenvironment. Bone marrow adipocytes act directly on other cell types in the bone marrow and secrete cytokines and adipokines that regulate bone remodeling.^70^ A growing body of research indicates that abnormal expansion of bone marrow adipose tissue is critical for the development of postmenopausal osteoporosis.^70^ In addition, bone marrow adipocyte senescence leads to secondary senescence of the bone marrow, which in turn leads to bone loss and osteoporosis.^71^ Recent evidence suggests that lipids or their intermediary metabolites are capable of influencing bone marrow adipocyte-related functions. Liu et al^71^ found that lipoxides induced bone marrow adipocyte senescence through activation of the PPARγ signaling pathway in a glucocorticoid-induced bone loss model. Senescent bone marrow adipocytes acquired the senescence-associated secretory phenotype, which spread senescence to bone marrow vascular cells and osteoblasts, leading to bone loss. Therefore, lipids could contribute to the development of osteoporosis by directly acting on bone marrow adipocytes, leading to senescence of bone marrow adipocytes or abnormal expansion of adipose tissue (Table 1).

Macrophages are bone marrow-derived mononuclear phagocytes that change their function and phenotype in response to different signaling stimuli in the bone microenvironment.^72^ Macrophages can be categorized into M1 and M2 subtypes. M1 macrophages are pro-inflammatory cells, and M1 macrophage-associated cytokines (such as tumor necrosis factor-α, interleukin-6, and IL-1β) induced osteoclastogenesis which promotes bone resorption.^72^, ^73^, ^74^ Thus, different polarization states of macrophages have an important role in bone homeostasis. Recent studies have shown that disorders of lipid metabolism lead to disturbances in macrophage function.^75^ Liu et al^76^ demonstrated that decreased levels of macrophage autophagic flux led to impaired autophagy in high-fat diet-induced obese mice. Inhibition of autophagy-related 5 expression polarized macrophages toward M1 phenotypes, leading to a systemic and tissue inflammatory response. Thus, lipids interfere with macrophage polarization, leading to an inflammatory response in the bone microenvironment and influencing bone remodeling (Table 1).

Bone is a highly vascularized organ.^77^ Vascular endothelial cells form the inner layer of the vascular system and act as a barrier for the transportation of substances between blood vessels and bone cells.^78^ It has also been found that blood vessels play an important role in the regulation of bone formation. A variety of cell-secreted factors enter the bone marrow microenvironment and promote the formation of type H vessels which produce factors that stimulate the proliferation and differentiation of bone histiocytes and positively direct bone formation.^79^ Therefore, angiogenesis is essential for bone development, bone regeneration, and bone remodeling.^80^ It has been shown that lipids, especially oxidized lipids, can damage vascular endothelial cells, leading to alterations in the structural integrity and function of the endothelial barrier. Yang et al^81^ demonstrated that in rat-derived endothelial progenitor cells with oxidized low-density lipoproteins' stimulation mimicking hypercholesterolemia condition, oxidized low-density lipoproteins activated autophagic fluxes of endothelial cells, inhibited the proliferation of endothelial progenitor cells, and impaired angiogenesis. The oxidized low-density lipoproteins increased endothelial cell stromal interaction molecule 1 expression and intracellular Ca^2+^ concentration.^81^ Therefore, lipid metabolism affects angiogenesis by influencing endothelial function, and impairing bone vascularization could lead to the development of a number of bone-related diseases, such as osteoporosis (Table 1).^80^

In conclusion, lipids, mainly oxidized lipids and cholesterol, promote lipogenic differentiation of MSCs and inhibit differentiation of osteoblasts. Besides, oxidized lipids and cholesterol can also upset the balance of bone remodeling, increase osteoclast activity, and decrease osteoblast activity. These changes ultimately increase bone marrow fat deposits, reduce bone formation, and promote bone resorption and the development of osteoporosis.

## Strategies for regulating lipids to treat osteoporosis

### Diet and exercise

Exogenous lipids are crucial for the development of osteoporosis. Therefore, in addition to pharmacological treatment, lifestyle modifications, dietary changes, and reasonable exercise can be used to treat osteoporosis or prevent further osteoporotic fractures.^82^ Regarding diet, it is important to reduce the intake of high-fat foods and low calcium intake, and high sodium, alcohol, and cola intake can decrease bone mass.^83^ Results of a trial evaluating the effects of low-fat low-carb diets on obesity and lipids in 6499 adults showed that low-fat low-carb diets were effective in reducing body weight and improving levels of HLD and triglycerides.^84^ Therefore, current research on diet and bone status encourages a balanced diet, including plenty of fruits and vegetables, adequate dairy products and other protein foods, and limiting foods with low nutrient density.^85^ Moreover, it is crucial to maintain moderate exercise and maintain a normal weight. Lipids are considered an important source of energy during exercise, mainly referring to the oxidation of fatty acids.^86^ Fatty acids may be signaling candidates for regulating the transcription of target genes encoding proteins involved in muscle lipid metabolism.^87^ As obesity is characterized by impaired fatty acid oxidation, fatty acid oxidation capacity is improved in obese individuals during exercise training. Thus, moderate exercise is a potential approach to improve the flexibility of lipid metabolism in obese patients.^87^ Moreover, Baek et al^88^ examined MGAT1 and hepatic adipogenic gene mRNA levels in high-fat diet-induced rats subjected to 6 weeks of regular aerobic exercise. The results showed that aerobic exercise inhibited the MGAT1 pathway, thereby ameliorating high-fat diet-induced NAFLD in obese rats. In addition, numerous studies have shown that exercise therapy can improve quality of life, such as physical function and action, pain, and vitality in postmenopausal women with osteoporosis or osteopenia.^89^ This shows that diet and exercise have an important and positive role in regulating lipid metabolism and preventing osteoporosis.

### Investigational agents targeting lipid metabolism and bone formation

Pathological conditions such as NAFLD, atherosclerosis, and obesity can alter the body's lipid metabolism, affecting bone mass and density and leading to osteoporosis. All these studies concluded that lipid metabolism disorders increase the chance of osteoporosis. Therefore, the use of lipid-lowering agents to regulate lipid metabolism in the body may have a role in treating and mitigating osteoporosis (Table 2).

**Table 2:** Potential agents for the treatment of osteoporosis targeting lipid metabolism disorders.

Agents	Effects	Mechanism	References
Statins	Lower plasma cholesterol levels, especially low-density lipoprotein cholesterol; promote bone anabolism and decrease bone resorption	Inhibit the activity of HMGCR; regulate the sterol regulatory element binding protein-2 pathway; up-regulate bone morphogenetic protein-2 and protect osteoblasts from apoptosis; reduce RANKL/OPG ratio and inhibit osteoclast differentiation	90,92,93
Metformin	Regulate blood sugar and lipid; restore damaged bone structure and bone density	Up-regulate expression of osteogenic marker genes; decrease OPG/RANKL ratio and inhibit osteoclast differentiation	94, 95, 96
Vitamin C/E, PCSK9 inhibitors	Reduce the production of oxidized lipids; slow down atherosclerosis	Neutralize free radicals and remove hydroxyl free radicals; scavenge lipid peroxidation free radicals; reduce tissue oxidative damage	98,99
PCSK9 inhibitors	Lower plasma lipid level and reduce the intake of exogenous lipids by bone cells	Inhibit PCSK9 expression/function; increase LDLR expression in liver	101,102
Saroglitazar	Lose weight; improve alanine aminotransferase level in nonalcoholic fatty liver disease; improve blood glucose and lipid parameters in atherosclerosis	Activate the PPARγ signaling	103,104
LT175	Lose weight; lower the levels of blood sugar, cholesterol, insulin, and triglycerides	Interact with a new recognition region of PPARγ	105
Bisphenol A diglycidyl ether	Enhance osteogenesis and inhibit fat formation	Inhibit the PPARγ signaling in mesenchymal stem cells	106
SR1664	Enhance osteogenesis and inhibit fat formation	Block obesity-induced phosphorylation of S273; inhibit the PPARγ pathway	107

Notes: HMGCR, HMG-CoA reductase; RANKL, receptor activator of nuclear factor-kappa B ligand; PCSK9, pro-protein convertase subtilisin/kexin 9; LDLR, Low-density lipoprotein receptor; OPG, osteoprotegerin; PPARγ, peroxisome proliferator-activated receptor gamma.

#### Statins

As a lipid-lowering agent, statins can inhibit the activity of HMG-CoA Reductase (HMGCR), effectively reducing plasma cholesterol levels, especially LDL-C.^90^ Low plasma cholesterol levels can increase hepatic LDL receptor (LDLR) expression through the sterol regulatory element binding protein-2-dependent pathway, accelerating the uptake and clearance of LDL-C from plasma.^90^ Statins seem to be a promising drug for the treatment of osteoporosis.^91^ According to the present literature, statins' effects on bone may entail several pathways. In addition to their lipid-lowering properties, statins exert their anabolic effects on bone by up-regulating bone morphogenetic protein-2, protecting osteoblasts from apoptosis, and controlling MSCs' commitment to osteoblasts.^92^ Furthermore, statins can increase and decrease the expression levels of OPG and RANKL mRNA in osteoclasts, respectively, reducing bone resorption by decreasing the differentiation and activity of osteoclasts through the OPG/RANKL signaling pathway.^93^ Thus, statins act as a primary agent to treat cardiovascular disease and thus regulate lipid levels and alleviate osteoporosis. Furthermore, it could play a vital role in the regulation of bone resorption as well as the maintenance of bone metabolism for the therapeutic management of osteoporosis.

#### Metformin

Metformin, an antidiabetic drug, promotes osteoblast differentiation, inhibits osteoclast differentiation, and prevents bone loss in de-ovalized rats.^94^ Studies have shown that metformin can increase alkaline phosphatase activity and expression of osteogenic marker genes at the mRNA and protein levels.^95^ It may act through multiple mechanistic effects to achieve therapeutic effects in osteoporosis. Nirwan and Vohora^96^ used a high-fat diet to feed C57BL/6 mice for 22 weeks to induce diabetic osteoporosis and treated with oral linagliptin and metformin. They measured femoral and tibial bone microarchitecture and BMD and examined histopathological changes. In addition, bone transformation biomarkers such as bone morphogenetic protein-2, sclerostin, anti-tartrate acid phosphatase, osteocalcin, and alkaline phosphatase were evaluated. Ultimately, the combination of linagliptin and metformin significantly restored damaged bone structure and density and positively modulated bone turnover biomarkers. In addition, Mai et al^94^ established that metformin dose-dependently stimulated OPG in mouse cranial and MC3T3-E1 osteoblasts and decreased RANKL mRNA and protein expression. Therefore, metformin could be used to treat high-glucose and high-fat-induced osteoporosis.

### Oxidized lipid antagonists

Numerous studies have established that oxidized lipids adversely impact lipid metabolism in bone, and lipid peroxidation is associated with various diseases and aging. Consequently, oxidized lipid antagonists may be used to prevent and counteract the negative effects of oxidized lipids on bone. Antioxidants can slow the progression of arterial thickening, atherosclerosis, and coronary heart disease.^97^ Vitamins E and C are typical lipophilic and hydrophilic antioxidants, respectively, and vitamin C scavenges free radicals in the aqueous phase. Furthermore, vitamin C intake reduces the effect of high high-cholesterol diet on alveolar bone density and osteoclast differentiation by reducing oxidative tissue damage by neutralizing free radicals and scavenging hydroxyl radicals.^98^ Its antioxidant activity may be clinically useful in hyperlipidemic patients to prevent alveolar bone resorption. Moreover, vitamin E scavenges lipid peroxide radicals.^99^ As a result, consuming certain doses of vitamins C and E can be used to intervene in the oxidative alteration of lipids. However, due to the diversity of lipid oxidation production pathways, including enzymatic oxidation, non-enzymatic free radical-mediated oxidation, and non-enzymatic non-free mediated oxidation,^99^ different countermeasures are required for oxidized lipids produced by different mechanisms, necessitating further exploration and researches.

### Drugs for regulating lipid transport

LDL influences the action of cholesterol on osteoclasts and osteoblasts.^100^ Perhaps it is feasible to modulate exogenous lipid uptake by blocking lipoprotein receptors on the surface of osteocytes or boost hepatic LDLR expression to reduce plasma LDL-C for changes in bone mass caused by excessive cholesterol or other lipids. Okayasu et al^101^ used LDL knockout (*LDLR*^*−/−*^) mice to elucidate the role of LDLR in regulating osteoclast differentiation and showed that bone mass was consistently increased in *LDLR*^*−/−*^ mice, accompanied by a decrease in bone resorption parameters, while bone formation parameters were unchanged. Pro-protein convertase subtilisin/kexin 9 (PCSK9) is a serine protease of the proprotein convertase family, mainly produced by the liver and up-regulates plasma LDL-C levels by inhibiting hepatic LDLR recirculation to the cell surface.^102^ Thus, PCSK9 inhibitors could increase hepatic LDLR expression by attenuating PCSK9 expression/function, thereby reducing plasma LDL-C. Accordingly, PCSK9 inhibitors are potentially valuable in treating cardiovascular disease and may further mitigate the development of osteoporosis by lowering lipid levels and regulating cholesterol metabolism.

### Inhibitors of PPARγ signaling pathway

PPARγ is a major regulator of lipogenesis and a very critical target in MSCs to determine their differentiation direction. Therefore, regulating the activity of PPARγ can regulate lipid levels and thus osteoporosis due to dyslipidemia and disorders of lipid metabolism. Saroglitazar is a novel agonist of PPARγ that improves glucose lipid parameters and insulin sensitivity. In a randomized controlled clinical trial, Gawrieh et al^103^ found that saroglitazar (4 mg) significantly improved alanine aminotransferase levels and atherosclerotic dyslipidemia in patients with NAFLD/NASH. In addition, Kumar et al^104^ verified the effect of saroglitazar on improving NASH by establishing a high-fat diet-induced NAFLD model in mice. The results also confirmed that saroglitazar treatment resulted in lower body weight, lower levels of total cholesterol, triglycerides, and alanine aminotransferase, and remission of NASH in mice. LT175 is a novel PPARγ ligand that interacts with a novel recognition region of PPARγ. It was shown that *in vivo* administration of LT175 to mice on a high-fat diet reduced body weight, adipocyte size, and white adipose tissue mass. In addition, LT175 significantly reduced blood glucose, cholesterol, insulin, triglycerides, and non-esterified fatty acids.^105^

In addition, inhibition of PPARγ transcriptional activity in MSCs to enhance osteogenesis and inhibit lipogenesis is also an effective means to alleviate osteoporosis. Li et al^106^ studied the effects of bisphenol A diglycidyl ether, an antagonist of PPARγ, on an OVX rat model and showed that early treatment with a 30 mg/kg dose of bisphenol A diglycidyl ether reduced bone marrow obesity and stimulated bone formation in ovary-intact and OVX rats. Marciano et al^107^ reported a PPARγ antagonist SR1664, which blocks obesity-induced serine 273 phosphorylation in the absence of classical agonists. Furthermore, they demonstrated that the inhibitory effect of SR1664 on PPARγ promotes osteogenic differentiation in MSCs and is consistent with the ideal bone phenotype observed in an animal model of PPARγ deficiency. Thus, it is evident that PPARγ is a critical regulatory target in osteoporosis triggered by dyslipidemia and lipid metabolism disorders.

## Conclusion and prospect

The results of clinical and basic studies revealed that lipids and lipid metabolites significantly influence the progression of osteoporosis. Results from *in vivo* experiments in animals suggested that lipid metabolism is directly related to the differentiation of MSCs and the homeostasis of bone metabolism. This review discussed the effects of NAFLD, atherosclerosis, and obesity on the occurrence of osteoporosis and attempted to reveal the cellular and molecular mechanisms of oxidative lipids and cholesterol causing osteoporosis. However, because lipid metabolism involves multiple organs and cells and has complex regulatory mechanisms, including many steps of lipid absorption and transport, the relationship between different types of lipids and their metabolism and the development of osteoporosis remain to be further elucidated. Furthermore, the origins of lipid metabolism abnormalities and their relationships with inflammation and other substance metabolism should be addressed, followed by a thorough examination of the mechanisms impacting bone metabolism.

In addition to descriptive investigations, we performed cellular and mechanistic explorations as new therapeutic targets for treating osteoporosis. This review shows that lipid metabolism disorder promotes the differentiation of MSCs toward lipogenic pathways and inhibits osteogenic differentiation. Besides, lipids affect bone homeostasis between osteoblasts and osteoclasts. As a result, in terms of therapeutic strategy development, osteoporosis can be alleviated by regulating lipids, and new therapeutic drugs can be developed based on cellular and molecular mechanisms. Due to the complexity of lipid metabolism, the mechanisms of lipid impacting osteoporosis need to be further explored; therefore, new therapeutic targets for osteoporosis need to be further discovered and studied. In conclusion, increasing evidence supports the close association of lipids and lipid metabolism with the development of osteoporosis, providing new insights that can contribute to the safe and rational selection of drug therapies to prevent and treat these common diseases.

## Conflict of interests

The authors declare no conflict of interests.

## Funding

The study was sponsored by the Key Program of the National Natural Science Foundation of China (No. 81930067), the 10.13039/501100001809Natural Science Foundation of China (No. 82002316), and the Youth Cultivation Project of Army Medical University (China) (No. 2020XQN08).
